# Children With Inflammatory Bowel Diseases are Disadvantaged by Current Drug Approval Policies: A Call for Urgent Change

**DOI:** 10.1093/crocol/otaf036

**Published:** 2025-06-19

**Authors:** Benjamin Sahn, Ross M Maltz, Joel R Rosh

**Affiliations:** Northwell, New Hyde Park, NY, USA; Division of Pediatric Gastroenterology, Liver Diseases, & Nutrition, Cohen Children’s Medical Center, New Hyde Park, NY, USA; Division of Pediatric, Gastroenterology, Hepatology, & Nutrition, Nationwide Children’s Hospital, Columbus, OH, USA; Department of Pediatrics, The Ohio State University Wexner Medical Center, Columbus, OH, USA; Northwell, New Hyde Park, NY, USA; Division of Pediatric Gastroenterology, Liver Diseases, & Nutrition, Cohen Children’s Medical Center, New Hyde Park, NY, USA

## Abstract

Treatment of pediatric inflammatory bowel disease (IBD) is significantly hindered by the lack of US Food and Drug Administration-approved biologic and small-molecule medications. This review explains the burdens faced by children with IBD because of this problem and appraises the marked historical timeline differences in medication approval between adults and children with IBD. The authors follow with an in-depth focus on the pointed disparity in approved therapies for children with IBD compared to children with rheumatologic immune-mediated diseases, highlighting the differences in stringency of evidence that has been used to gain medication approval for children with rheumatologic diseases. The editorial concludes with a call for change in regulatory agency protocols, to adopt a modernized strategy that will expedite the approval of advanced therapies for children with IBD.

The divide between the number of advanced therapies approved to treat children with inflammatory bowel disease (IBD) compared to adults has grown into a chasm in the United States. While there are 2 advanced therapies within one drug class approved by the US Food and Drug Administration (FDA) for children, adults with IBD now have 13 FDA-approved advanced therapies spanning six different mechanistic targets (**Table 1**). The result of this disparity has been a chaotic treatment landscape for pediatric gastroenterologists filled with insurance company denials. Rather than optimized care, the current state leads to limited treatment options for children with IBD based on the absence of FDA approval. Delays in initiating therapy and approval of clinically inadequate dosing are common, leading to increased disease activity and potential loss of drug response. This results in harmful events, poor patient outcomes, and extended hours of additional work by medical staff appealing insurance denials for coverage.^1^

**Table 1.** FDA-approved medications for adults and children with IBD.

Adults ≥ 18 years	Pediatrics 5 to ≤ 17 years	Pediatrics < 5 years
**Anti-TNF** Infliximab Adalimumab Certolizumab—pegol Golimumab **Anti-Integrin** Vedolizumab **Anti IL-12/23** Ustekinumab **Anti IL-23** Risankizumab-rzaa Mirikizumab-mrkz Guselkumab **JAK-Inhibitor** Upadacitinib Tofacitinib **S1P Agonist** Ozanimod Etrosimod	**Anti-TNF** Infliximab (≥ 6 years) Adalimumab (≥ 5 years)	None

Listing is by originator agent.

TNF, tumor necrosis factor; JAK, Janus Kinase; S1P, sphingosine 1 phosphate receptor.

A common response to the current state is the off-label use of advanced agents in children with IBD despite sparse pharmacokinetic (pK) and pharmacodynamic (pD) data to guide dosing. While this reality may be preferred to corticosteroid dependency, hospitalization, and/or surgical resections, it has further strained the clinician’s ability to enroll pediatric patients into pivotal phase III, randomized, clinical trials that are initiated long after adult approval. It is difficult to argue for equipoise between obtaining off-label medication and the uncertainty of receiving a non-optimized and potentially lower than therapeutic dose of an investigational product in an underpowered clinical trial. The pool of available patients willing to participate in a clinical trial program is then further diminished by unrealistic protocol designs for children requiring long medication washout periods and invasive procedures, leaving an already vulnerable population at greater risk of disease complications or emotional and physical distress. Recently published FDA guidance did not offer a new pathway to drug approval in pediatric IBD and several pediatric and adult gastroenterological societies along with pediatric IBD authorities pushed back, sounding the alarm on the perpetual cycle of inferior treatment options for children with IBD and the need to change the requirements for FDA drug approval for pediatric IBD.^2,3^

The ongoing absence pediatric label for vedolizumab (VDZ) is representative of the current regrettable state. Approved more than 10 years ago for adults with IBD, there is still no pediatric indication despite a significant body of real-world evidence demonstrating efficacy in children. Ultimately, enormous resources were utilized to conduct a controlled trial evaluating induction and maintenance of remission in children, requiring 17 medical centers in 6 countries to enroll (only) 137 children over 6 years.^4^ This herculean and applaudable undertaking included crucial pK/pD analyses, which have greatly advanced our knowledge of VDZ dosing in children.^5^ Valuable years could have been saved if the pK/pD studies were allowed to be the primary focus of a targeted study to optimize VDZ use in children, and approval were given based upon extrapolation of efficacy from adult studies. Ustekinumab has a similar history to that of vedolizumab, quickly approaching a decade since approval in 2016 for adults with CD.

A lengthy time to pediatric IBD approval has been the timeline since the introduction of infliximab to the drug market in 1998 for adults with Crohn’s disease. Eight years were needed before the REACH trial led to pediatric CD approval,^6^ and there was a 6-year delay for pediatric ulcerative colitis (UC), which ended in 2011. Adalimumab approvals were similarly delayed, with the IMAgINE 1 trial leading to pediatric CD approval in 2014, 7.5 years after adult approval, and the ENVISION I trial ushering pediatric UC approval in 2021, which was 9 years behind the adult approval for UC.^7,8^

As disheartening as the lack of advanced therapies FDA-approved for children with IBD proves to be, it is even more surprising to recognize the number of advanced therapies with FDA approval for *children* with rheumatologic (**Table 2**) and other immune-mediated inflammatory diseases. Notably, several of these therapies were approved with a less stringent body of evidence than what is required for approval in children with IBD. As examples, golimumab received FDA approval in 2020 for children 2-17 years with juvenile inflammatory arthritis (JIA) based on an open-label trial treating 127 subjects for 52 weeks.^9^ Pharmacokinetic exposure was a major endpoint and found to be similar in adults and children. These results also led the FDA to extend the label to children with juvenile psoriatic arthritis (JPsA), while only five children in the study had JPsA. Ustekinumab gained approval in 2022 for the treatment of JPsA based on extrapolation of efficacy and pK data from randomized, placebo-controlled trials in adults and children with plaque psoriasis and adults with psoriatic arthritis.^10,11^ After well controlled trials led to approval of intravenous tocilizumab for systemic and polyarticular JIA, FDA approval for the subcutaneous formulation was received based on 52-week open label, phase 1b pK/pD studies enrolling 52 and 51 patients, respectively, that tested a range of doses along with extrapolation of efficacy from adults with rheumatoid arthritis.^12^ Upadacitinib, the most recent advanced therapy to be approved for JIA and PsA, received its approval in 2024 utilizing extrapolation of efficacy from well-controlled trials in adults with rheumatoid arthritis and psoriatic arthritis, along with pK data in 51 children with JIA participating in an open-label phase 1 trial.^13^ Golimumab, ustekinumab, and upadacitinib are all efficacious in adults with IBD and considered safe in children with rheumatologic disease, but remain without approval for pediatric IBD. In all, the FDA has shown a willingness to accept a model of extrapolation of efficacy from adult data coupled with early pK analysis in children with rheumatologic disease. Unfortunately, FDA approval for children with IBD is kept to a different standard, which is leading to significant patient harm. The FDA approval process for advanced therapies used to treat children with IBD needs to change, or the care of this patient population will continue to be hampered.

**Table 2.** FDA-approved advanced therapies for pediatric rheumatologic diseases.

Drug	Drug class	Indication for pediatrics	Year of FDA approval	Phase III placebo-controlled clinical trial in pediatric disease receiving FDA approval labeling
Abatacept	Anti TNF	JIA JPsA	2008 2023	Yes* No
Adalimumab	Anti TNF	JIA	2008	Yes*
Golimumab	Anti TNF	JIA JPsA	2020 2020	No No
Etanercept	Anti TNF	JIA JPsA	1999 2023	Yes* No
Tocilizumab	Anti IL-6	SJIA pcJIA pcJIA SJIA	2011 2013 2018 2018	Yes* (IV formulation) Yes* (IV formulation) No (SC formulation) No (SC formulation)
Canakinumab	Anti IL-1b	JIA	2013	Yes
Secukinumab	Anti IL-17a	JPsA ERA	2021 2021	Yes Yes
Tofacitinib	JAK inhibitor	pcJIA	2020	Yes*
Upadacitinib	JAK inhibitor	pcJIA JPsA	2024 2024	No No
Ustekinumab	Anti IL-12/23	JPsA	2022	No

*Placebo controlled in maintenance phase. Trials designed as open label medication for induction of remission followed by randomization to medication or placebo (medication withdrawal) for maintenance.

JIA, Juvenile inflammatory arthritis; SJIA, Systemic juvenile inflammatory arthritis; pcJIA, polyarticular course juvenile inflammatory arthritis; JPsA, juvenile psoriatic arthritis; ERA, enthesitis related arthritis; TNF, tumor necrosis factor; JAK, Janus Kinase; IV, intravenous; SC, subcutaneous.

To move the treatment of pediatric IBD forward, a paradigm similar to that used in pediatric rheumatologic disease needs to be embraced. By starting pediatric trials before regulatory authorization for adults is in place, designing adult clinical trial programs to optimize extrapolation of efficacy for pediatric patients, mandating post-marketing safety registries in children, and most critically creation of pediatric focused pK/pD studies (inclusive of our smallest patients <30 kg) in the early stages of drug development, a clear and achievable strategy would emerge.^2,14^

The current status quo is harming children with IBD and cannot continue. The pediatric gastroenterology community, the Crohn’s & Colitis Foundation, and other advocacy groups need to be heard. It is time that regulatory agencies support this with imperative policy shifts that will quickly open doors to therapies for children with IBD.

## Data Availability

No new data was created or analyzed in the production of this work.
